# Effect of polymer rigidity on the phase behaviour of polymer adsorption on to planar surface

**DOI:** 10.1042/BSR20160220

**Published:** 2016-11-22

**Authors:** Zhiyong Yang, Aihua Chai, Peicong Zhou, Ping Li, Yongfu Yang

**Affiliations:** * Department of Physics, Jiangxi Agricultural University, Nanchang, Jiangxi 330045, China; † College of Mathematics, Physics and Information Engineering, Jiaxing University, Jiaxing, Zhejiang 314001, China

**Keywords:** Monte Carlo method, semiflexible polymer, toroidal structure

## Abstract

We study the process of a semiflexible polymer chain adsorption on to planar surface by the dynamic Monte Carlo (DMC) method, based on the 3D off-lattice model. Both the strength of attractive monomer–surface interaction (*ε*_a_) and bending energy (*b*) have pronounced effect on the adsorption and shape of semiflexible polymer chain. The semiflexible polymer can just fully adsorb on to the surface at certain *ε*_a_, which is defined as critical *ε*_a_. The essential features of the semiflexible polymer adsorption on to surface are that (i) the critical *ε*_a_ increases with increase in *b*; (ii) the shape of the fully adsorbed semiflexible polymer chain is film-like toroid, and the toroid becomes more and more perfect with increase in *b*. In addition, the size of toroid and the number of turns of toroid can be controlled by the *b* and *ε*_a_.

## INTRODUCTION

The problem of polymer adsorption on to surfaces is relevant in many contexts, such as control of nanocrystal growth by adsorbed polymers, biomolecules interaction with cell surfaces, fabrication of special polymeric coatings at substrates and so on. Therefore, understanding the properties of polymers near a surface or interface is important in polymer and biology sciences. The polymer in solution placed in contact with a surface can readily adsorb on to various surfaces if there exists an attractive interaction between segments of polymer and surface, and it can overcompensate for the conformational entropy loss associated with the adsorption [1]. Such an adsorption process may be governed by properties of the surface, the polymer or the solvent as well as the fine interplay among these. The polymer adsorption on to surface is very fundamental in a wide range of applications. Moreover, understanding and controlling such processes is of great importance and is essential in many different technological aspects ranging from paper industry and paint formulation to pharmaceutical applications [2], biophysics [3–5] and nanocomposite materials [6]. As we know, the conformation of polymer changes greatly in the process of binding to surface. The conformational changes caused by the adsorption process can trigger drug delivery, enzymatic catalysis or cellular motion.

It is very interesting to understand how the conformational changes that a polymer can experience in the adsorption process to surface are largely governed by the prevailing conditions under which polymer, solvent and surface interact. Obtaining detailed information on the conformation and dynamics of polymers on to surface can greatly aid the development of new polymer materials, chemical industry and medical science.

Based on the practical application, the topic of adsorption has received intensive experimental [7–13], theoretical [14–19] and simulative [20–26] attention. Previously, there is a growing interest in adsorption process of biopolymers such as RNA/DNA and proteins on to solid surface, which may reveal important information on conformation, function and kinetics of RNA/DNA and protein [27,28]. It is well known that intrinsic chain stiffness is a very important characteristic of biopolymer [29–33]. For semiflexible polymers, the stiffness of a semiflexible polymer is intermediate between random coils and rigid rods. Many biopolymers such as DNA, filamentous (F-) actin or microtubules belong to the class of semiflexible polymers. Kierfeld et al. [14] show that the adsorption threshold of semiflexible polymers on to a planar substrate can be controlled by polymer stiffness for polymer grafted one chain end to surface, and the adsorption threshold can be additionally controlled by the curvature of curved substrates. Källrot et al. [20] find that the semiflexible chains that fully adsorb on to surface exhibit a flatter conformation as compared with the flexible chain for short chain. Möddel et al. [21] construct the pseudophase diagram of thermodynamic conformational phases of a single semiflexible homopolymer near an attractive substrate in dependence of the attractive monomer–surface interaction and temperature for short polymer chain. They find that the fully adsorbed polymer takes on circularly compact film-like shape. Ivanov et al. [26] find single semiflexible chain tethered to a planar surface of a long-ranged attractive potential can fully adsorb on to surface and form film-like ordered structure for short chain. In the past, scientists pay more attention to the process of short semiflexible chain adsorption on to surface, and the chain is tethered to surface. In the present study, we characterize the adsorption process of semiflexible polymer chain near an attractive surface for long polymer chains using dynamic Monte Carlo (DMC) simulation. The present study aims at systematically investigating the effect of chain stiffness and attractive polymer–surface interaction on the adsorption process. Our research findings show that the fully adsorbed conformations of long semiflexible polymers are different from that of short semiflexible polymers.

## MATERIALS AND METHODS

A simple coarse-grained model is used to investigate the structural behaviour of single semiflexible polymers adsorption on to planar surface. The polymer is represented by a chain of *N* + 1 spherical beads connected via the finitely extendable nonlinear elastic (FENE) potential (where *N* is the chain length of polymer) [34].

1 $$U_{FENE}=-k{r_{0}}^{2}\ln\left[1-{\left(\frac{l_{i}-l_{0}}{r_{0}}\right)}^{2}\right]$$

In eqn (1), *l_i_* is the length of *i*th effective bond, which can vary in the range of *l*_min _ < *l_i_* < *l*_max _ with *l*_min_=0.4 and *l*_max_=1.0, and its preferred distance *l*_0_ is 0.7(where *l*_max_ is the unit of length). *r*_0_=*l*_max _ − *l*_0_=*l*_0_ − *l*_min _ and the spring constant (*k*) is set to 20 in the unit of *k*_B_*T* (where *k*_B_ is the Boltzmann constant and *T* is the thermodynamic temperature). *k*_B_*T* is chosen to be the unit of energy.

Volume exclusion for all non-bonded monomers is imposed via a Morse-type potential [34].

2 $$\begin{array}{ccc} U_{M} & = & \sum_{\left|i-j\right|>1}ɛ\;(\exp\left(-2\alpha \left(r_{ij}-r_{\min}\right)\right)\\ & & -\;2\exp(-\alpha \left(r_{ij}-r_{\min}\right))) \end{array}$$

In eqn (2), *r_ij_* is the distance between the *i*th monomer and the *j*th monomer and α=24, *r*_min_=0.8 and ε=1 are selected. Owing to the large value of α, *U*_M_ decays to zero very rapidly for *r_ij_*>*r*_min_, and is completely negligible for distances larger than unit length. The combination of FENE bonds with excluded volume interactions is beneficial to prevent unphysical crossing of the polymers.

The stiffness of the chain is regulated by angular potentials [34].

3 $$U_{b}=b\left(1+\cos\theta \right)$$

In eqn (3), θ is the angle between two consecutive bonds, and *b* is the bending energy. *b* can be considered as a penalty for successive bonds deviating from a straight arrangement, i.e. the chain rigidity can be adjusted by varying *b*. In addition, *b* is in the unit of *k*_B_*T*.

The infinite planar surface is located at *z*=0. The semiflexible polymer chain is placed above the planar surface. The monomers of polymer chain interact with the surface by a Morse-type potential.

4 $$U_{S}=\sum_{}{ɛ}_{a}\left(\exp\left(-2\alpha \left(r_{i}-r_{\min}\right)\right)-2\exp\left(-\alpha \left(r_{i}-r_{\min}\right)\right)\right)$$

Here, *r_i_* is the distance of the *i*th monomer to the surface. *ε*_a_ defines strength of attractive monomer–surface interaction, and it can adjust the attractive interaction of polymer–surface.

**Figure 1:**
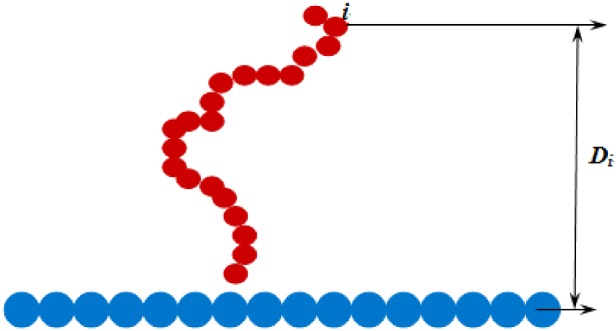
Sketch of the polymer adsorption on to planar surface Sketch of the polymer adsorption on to planar surface. The stripped red solid circle represents polymer. The stripped blue solid circle represents surface. Here, *D_i_* is the distance between *i*th monomer and surface.

The polymer is placed near the attractive surface, and the monomer closest to the surface is in attractive range of surface, as shown in Figure 1. *D_i_* is the distance of *i*th monomer to surface. Then, the adsorption process is simulated via DMC simulations [35]. DMC simulations are performed according to the Metropolis algorithm. In more detail, a monomer is chosen randomly. The randomly chosen monomer is displaced from its position (*x*, *y*, *z*) to a new position (*x*′, *y*′, *z*′), and increments Δ*x*=*x*′ − *x*, and Δ*z*=*z*′ − *z* are chosen randomly from the intervals of − 0.15 ⩽ Δ*x*, Δ*y*, Δ*z* ⩽ 0.15 respectively. A trial move is accepted if Δ > η, where Δ=min (exp [ − Δ*U*/*k*_B_*T*], 1) is the transition probability depending on the energy difference Δ*U* between the trial and old states and η is a number uniformly distributed in the interval [0,1]. *N* + 1 trial moves are considered as one Monte Carlo step (MCS). If the polymer diffuses far away from the surface, the simulation restarts again. We perform 100 independent runs for each set of parameters, and each independent run includes 100 measurements at intervals of 1 × 10^6^ MCS after the chains reach equilibrium. Therefore, we get 10000 samples. The statistical quantities of polymer chains are acquired by calculating the arithmetic mean of 10000 samples. The polymer chain length is set to *N*=300.

## RESULTS AND DISCUSSION

### The adsorption process

**Figure 2:**
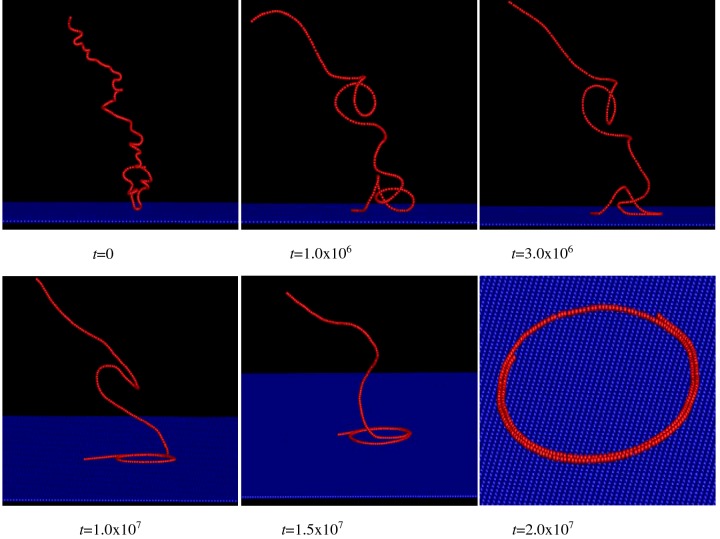
The conformation of the process of polymer adsorption on to planar surface The snapshots of configurations of semiflexible polymer for *b*=300 and *ε*_a_=4 at different times *t*.

The work pays more attention to the adsorption process of polymer translocation in biological body. Our major objective is to study the conformation of semiflexible polymer that fully adsorbs on to planar surface. Firstly, the process of adsorption on to surface is studied for semiflexible polymer. It is typical for the polymer to fold into toroid structure in the regime of moderate bending energy (*b*) and the attractive monomer–surface interaction (*ε*_a_). The probability of forming the toroid structure is over 80%. Therefore, a typical sample can be used to show the process of forming the toroid structure. The Figure 2 shows the snapshots of semiflexible polymer at different time *t* for *b*=300 and *ε*_a_=4. At *t*=0 MCS, the configuration is very much extended, and only one or two monomers are in the attractive range of surface. At *t*=1.0×10^6^ MCS, one end of polymer adsorbs on to surface. The polymer consists of one loop, two trains and one free long tail. At *t*=3.0×10^6^ MCS, one train becomes arc-like. At *t*=1.0×10^7^ MCS, the monomers that adsorb on to surface arrange into loop. Then, monomers of free tail adsorb on to the surface and wrap around the loop with time evolution. At last, the polymer folds into film-like toroidal structure at *t*=2.0×10^7^ MCS. Vanderlinden and Feyter [10] show that subchain that adsorbs on to plane can form a loop at the beginning, then the loop is reduced in size via an in-plane diffusive process, finally it disappears. The stiffness of chain drives the loop open. If strength of the attractive monomer–surface interaction is very large, relative position of monomers that adsorb on to the surface is very difficult to change. Therefore, it is very difficult to reduce the size of loop in the in-plane diffusive process.

**Figure 3:**
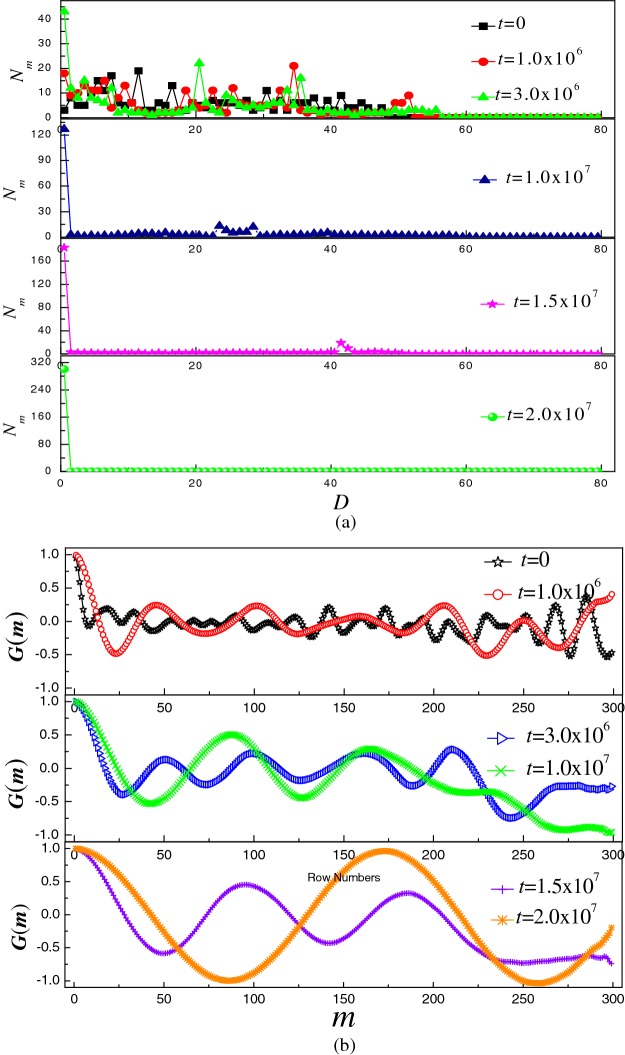
Monomers distribution and their spatial correlation function (**a**) *N*_m_ compared with *D* for the six configurations of Figure 2. Here, *D* is the distance between the monomer and the surface; *N*_m_ is the number of monomers in the range of *D*−0.5–*D*+0.5. (**b**) A spatial correlation function *G*(*m*) for six configurations of Figure 2.

Then, we study the distribution of monomers and the shape of polymer in adsorption process in detail. First of all, the distribution of monomers above the planar surface is studied for the six configurations of Figure 2, as shown in Figure 3(a). Here, *D* is the distance of the monomer to the planar surface and *N*_m_ is the number of monomers in the range of *D*−0.5–*D*+0.5. The distribution of monomers shows that although more and more monomers adsorb on to the surface, length of the free tail along the normal direction of surface increases with time in the initial adsorption process. It indicates that the free tail stretches itself along the normal direction of surface to adjust its position. If monomers of the free tail do not adjust their position, the chain will overlap on the surface. In addition, the chain is of rigidity. Therefore, it is easier for the free tail to stretch itself along the normal direction of surface. Then, the interplay among the bending energy, self-attractive energy of polymer and attractive energy of surface drives the polymer from toroid structure.

Next, a spatial correlation function *G*(*m*) is used to describe the shape of six configurations of Figure 2, as shown in Figure 3(b). The function can quantitatively distinguish between helical periodicity and coil [36].

$$G\left(m\right)=\frac{1}{N^{\prime }-3}\sum_{i=2}^{N^{\prime }-2}g(m,i)$$

Here, *N*′ is the number of monomers in the polymer chain, *m* means sequence interval. *g*(*m*,*i*) is given as

$$\begin{array}{ccc} & & g(m,i)\\ & & \;=\frac{1/\left(N^{\prime }-m-1\right)\sum_{j=1}^{N^{\prime }-m-1}(\cos\theta_{i,j}-\bar{\cos\theta_{i,j}})\left(\cos\theta_{i,j+m}-\bar{\cos\theta_{i,j}}\right)}{1/\left(N^{\prime }-1\right)\sum_{j=1}^{N^{\prime }-1}{\left(\cos\theta_{i,j}-\bar{\cos\theta_{i,j}}\right)}^{2}} \end{array}$$

The angle θ_*i*, *j*_is defined as

$$\cos\theta_{i,j}=\frac{{\overset{⇀}{l}}_{i}\cdot {\overset{⇀}{l}}_{j}}{\left|{\overset{⇀}{l}}_{i}\right|\left|{\overset{⇀}{l}}_{j}\right|}$$

Here, 
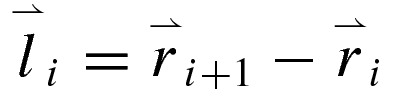
 is the *i*th bond vector, 
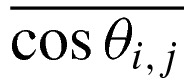
 denotes the average of cos θ_*i*, *j*_over *j* from 1 to *N*−1.

The *G*(*m*) for six configurations in Figure 2 is shown in Figure 3(b). It can provide evidence for toroidal configuration of semiflexible polymer. It can be observed that the *G*(*m*) oscillates randomly in the beginning, then *G*(*m*) takes on local periodicity and the periodicity becomes better and better with time, *G*(*m*) oscillates periodically in the end. Combining with the results of *N*_m,_ we can conclude that the semiflexible polymer can form film-like toroidal conformation.

### The effect of the attractive monomer–surface interaction and bending energy on the adsorption

As all we know, strength of the attractive monomer–surface interaction (*ε*_a_) is energetically favourable to form as many monomer–surface contacts as possible, whereas the bending energy (*b*) make the polymer less favourable to contact with surface. How do *ε*_a_ and *b* compete with each other in adsorption process? This is key point of study in this section.

**Figure 4:**
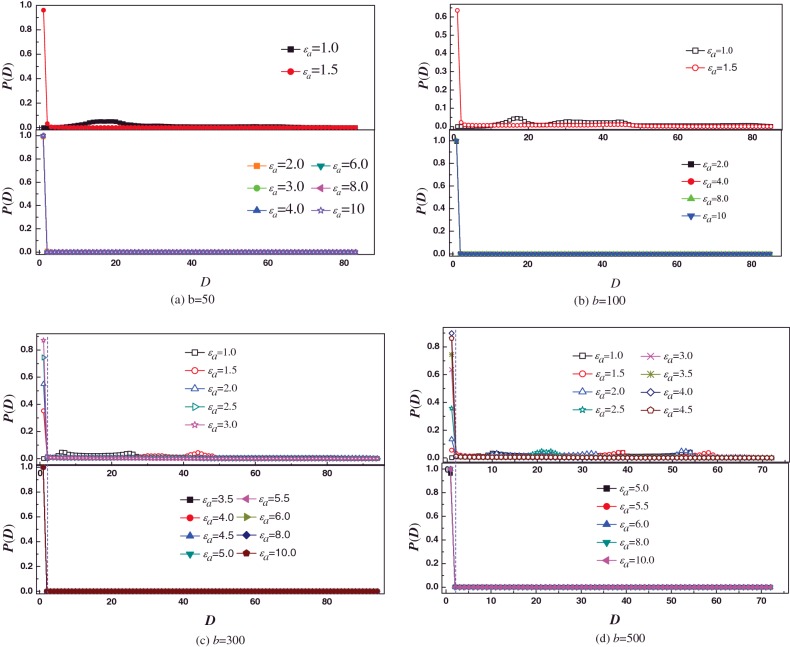
The effect of *b* on distribution of monomers along the normal direction of planar surface The distribution of monomers of the semiflexible polymer (**a**) *b*=50, (**b**) *b*=100, (**c**) *b*=300 (**d**) *b*=500 for different *ε*_a_.

At first, the effect of the attractive monomer–surface interaction (*ε*_a_) on the adsorption is studied. The distribution of monomers above the surface as a function of the distance *D* of the monomer to surface is shown in Figure 4. *P*(*D*) is the proportion of monomers in the range of *D*−0.5–*D*+0.5. For *b*=50, the *P*(*D*) of monomers binding to surface increases sharply from 0 to 0.93 with *ε*_a_ increasing from 1.0 to 1.5, and it almost reaches 1.0 with *ε*_a_ increasing further, as shown in Figure 4(a). For *b*=100, the *P*(*D*) of monomers binding to surface is only 0.64 for *ε*_a_=1.5. It increases to 1.0 quickly with increase in *ε*_a_, as shown in Figure 4(b). For *b*=300, the *P*(*D*) increases from 0 to 0.87 with *ε*_a_ increasing from 1.0 to 3.0, and reaches maximum at *ε_a_*=3.5, as shown in Figure 4(c). For *b*=500, the critical point appears at *ε*_a_=5.0, as shown in Figure 4(d). It indicates that the more rigid the polymer chain is, the more difficult the polymer chain binds to surface. It is in agreement with the results of Stepanow [37].

**Figure 5:**
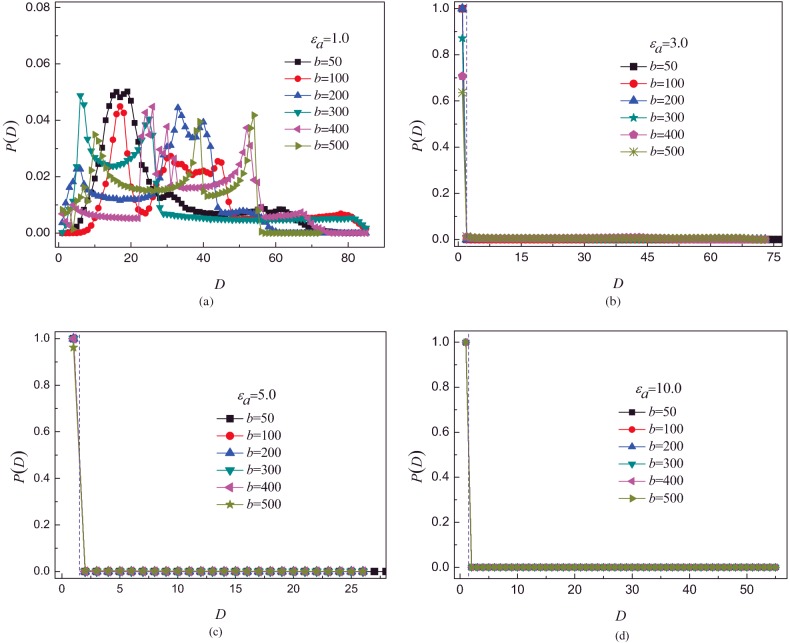
The effect of *b* on distribution of monomer along the normal direction of planar surface The distribution of monomer as a function of distance to surface *D* for different bending energy *b* at different surface attraction strength (**a**) *ε*_a_=1.0, (**b**) *ε*_a_=3.0, (**c**) *ε*_a_=5.0 (**d**) *ε*_a_=10.

Next, we study the effect of bending energy (*b*) on the distribution of monomer along the normal direction of surface, as shown in Figure 5. When the strength of attractive monomer–surface interaction (*ε*_a_) is very weak, although the polymer chains do not adsorb on to the surface for all *b*, the more and more monomers contact with surface with increase in *b*, as shown in Figure 5(a). It is known that the conformation of polymer is coiled for *b*=1, and the polymer chain becomes more and more extended with the increase in *b*. For *b*=50, the polymer collapses in the beginning, and the polymer is not very long. It leads that the size of polymer is not very large. In addition, *ε*_a_ is so small that increment of attractive energy does not compensate the entropy loss of polymer when the polymer adsorbs on to the surface. Therefore, the polymer diffuses away from the surface. The polymer stretches itself along the normal direction of surface, and becomes more and more extended with the increase in *b*, i.e. the size of polymer becomes larger and larger in the normal direction of surface. Therefore, it is easier for more rigid polymer to contact with surface. However, the entropy reduction is very large when the polymer adsorbs on to the surface. The attractive energy is not enough to compensate the entropy loss for *ε*_a_=1.0. For *ε*_a_=3.0, Figure 5(b) shows that semiflexible polymer fully adsorbs on to surface for *b*≤200, whereas the *P*(*D*) of monomers binding to surface decreases with increase in *b* for *b*>200. For *ε_a_*=5.0, Figure 5(c) shows that semiflexible polymer fully adsorbs on to surface for all *b*, except *b*=500, however, only about dozen monomers are out of attraction range of surface for *b*=500. When *ε*_a_ increases to 10.0, semiflexible polymer chain fully adsorbs on to surface for all *b*, as shown in Figure 5(d). It is intuitively clear that the more rigid the semiflexible polymer chain is, the larger external force that drives semiflexible polymer chain deform is. Therefore, in order to induce the polymer chain fully adsorbs on to surface, critical *ε*_a_ increases with *b*.

**Figure 6:**
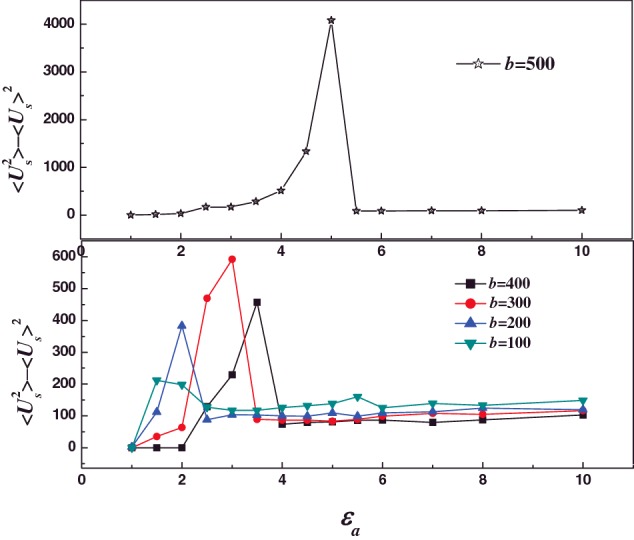
The total surface attraction energy fluctuation The fluctuation < *U*^2^_*s*_ > − < *U_s_* > ^2^ of semiflexible polymer chain plotted compared with surface attraction for different bending energy *b*. Here, *U*_s_ is the total surface attraction energy.

The specific heat *C*_v_ (
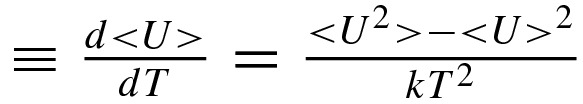
) is often used to characterize phase transition of polymer system [38]. The energy fluctuation < *U*^2^_*s*_ > − < *U_s_* > ^2^ is very similar to *C*_v_, where *U*_s_ is the total surface attraction energy. Therefore, < *U*^2^_*s*_ > − < *U_s_* > ^2^ can be used to characterize phase transition of semiflexible polymer chain. < *U*^2^_*s*_ > − < *U_s_* > ^2^ as a function of surface attraction energy *ε*_a_ is studied for different bending energy *b*, as shown in Figure 6. It can be observed that < *U*^2^_*s*_ > − < *U_s_* > ^2^ exhibits a pronounced peak for all *b*, and the position of the peak shifts from small *ε*_a_ to large *ε*_a_ with increase in *b*. The values of phase transition point are 2.0, 2.75, 3.5, 4.25, 5.0 for *b*=100, 200, 300, 400, 500 respectively. It is in agreement with the critical point of full adsorption, seeing the Figure 4. It further indicates that the semiflexible polymer chain shifts from desorbed conformation to fully adsorbed conformation with increase in *ε*_a_.

### The shape of adsorbed conformation

The shape has very pronounced effect on the property of polymer. The spatial correlations of the tangent vectors can be used to describe the structure of polymer [39]. The spatial correlation can be measured in experiments, and the shape of the polymer chain can be characterized by average tangent veoctor [40]. Therefore, the tangent–tangent correlation function *C*(*s*, *s*′) is used to study the shape of adsorbed polymer in the part. *C*(*s*, *s*′) is defined as

$$<\left(u\left(s\right)-<u\left(s\right)>\right)><\left(u\left(s^{\prime }\right)-<u\left(s^{\prime }\right)>\right)>$$

where **u**(*s*) and **u**(*s*′) are the tangent vectors at monomers *s* and *s*′ respectively. In our simulation, we measure *C*(*s*) ≡ *C*(*s*, 0) because of translational invariance and the indistinguishability of the two ends of polymer chain.

**Figure 7:**
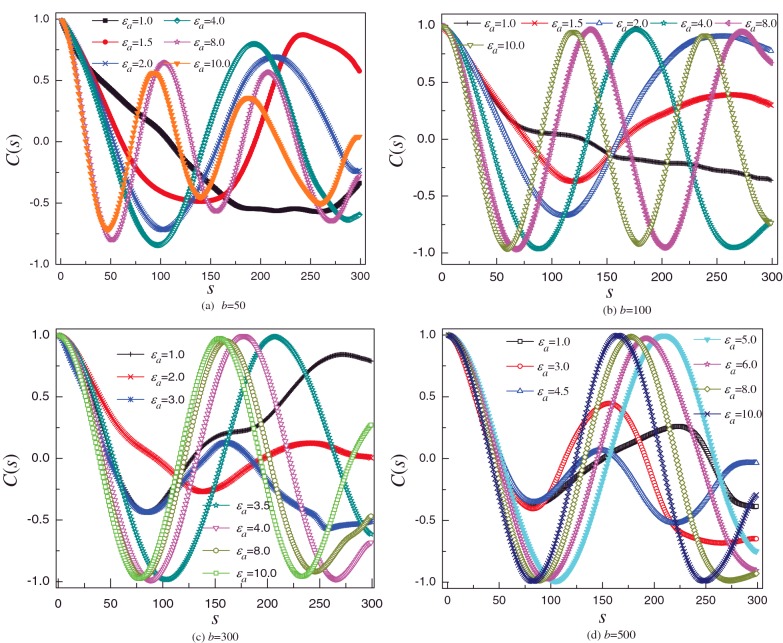
Tangent-tangent correlation *C*(*s*) for different condition Tangent–tangent correlation *C*(*s*) of semiflexible polymer under the condition of different surface attraction strength *ε*_a_ for (**a**) *b*=50, (**b**) *b*=100, (**c**) *b*=300 (**d**) *b*=500.

The Figure 7 shows that *C*(*s*) is measured at 1.0 ⩽ ε_*a*_ ⩽ 10 for different semiflexible polymer. For *b*=50, *C*(*s*) exhibits exponential decay for *ε*_a_=1.0, whereas it exhibits periodic oscillations, and the number of period increases with increase in *ε*_a_ forε_*a*_ ⩾ 1.5, as shown in Figure 7(a). Our previous study indicates that if *C*(*s*) exhibits periodic oscillations, the shape of polymer chain takes on helical structure and the number of period of *C*(*s*) is almost equal to the turns of helix [35]. We can infer that the attractive potential depth of planar surface is approximately 0.22, based on the eqn (4). Therefore, when the semiflexible polymer fully adsorbs on to the surface, its conformation is quasi 2D. The polymer chain fully adsorbs on to surface for *b*=50 when *ε*_a_ is greater than or equal to 1.5. Therefore, the conformation of polymer is quasi 2D toroidal structure and the number of turns of toroid increases with *ε*_a_ for ε_*a*_ ⩾ 1.5. For *b*=100, *C*(*s*) exhibits periodic oscillations for ε_*a*_ ⩾ 2.0, and the number of period increases with *ε*_a_, in addition, its periodic oscillations is better than that of *b*=50, as shown in Figure 7(b). *C*(*s*) of *b*=300 and *b*=500 also shows that *C*(*s*) exhibits perfect periodic oscillations for the fully adsorbed conformation. Comparing the four figures of Figure 7, it can be found that the number of period of *C*(*s*) decreases with increase in *b* for the same *ε*_a_. It is easy to understand that it is more and more difficult for polymer to bend with increase in *b*. Therefore, if the polymer chain folds into toroid, radius of toroid increases with *b* for the same *ε*_a_, i.e. the number of period of *C*(*s*) decreases with increase in *b*. *ε*_a_ plays the role of increasing the self-attractive strength of semiflexible polymer chain.

**Figure 8:**
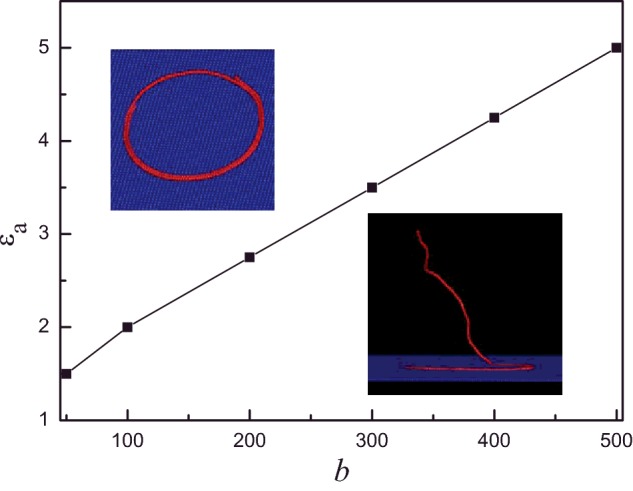
Diagram of states Diagram of states, surface attraction strength *ε*_a_ compared with bending energy *b.*

Based on the above results, both *b* and *ε*_a_ have pronounced effect on the shape of semiflexible polymer. Fully adsorbed conformation is key point of our study, therefore, the desorbed state and partially adsorbed state of semiflexible polymer chain are classified as the same state in the present study. The structural behaviours are summarized in the phase diagram, as shown in Figure 8. The semiflexible polymer is in two states: partially adsorbed and fully adsorbed. The representative conformations of the different phases are shown in the inset of Figure 8. The critical attractive monomer–surface interaction *ε*_a_ increases with *b*. It is in agreement with the results of Kong and Muthukumar [41].

## CONCLUSION

In the present study, we use DMC method to study the process of semiflexible polymer adsorption on to the planar surface, based on 3D off-lattice model. The purpose of our study is to clarify the effect of chain stiffness *b* and the attractive monomer–surface interaction *ε*_a_ on the adsorption of semiflexible polymer. Our study results show that both *b* and *ε*_a_ have pronounced effect on the adsorption and shape of fully adsorbed semiflexible polymer. The states of polymer can be roughly classified into partially adsorbed state and fully adsorbed states. The critical *ε*_a_ increases with *b*. We observe a very interesting structure for the fully adsorbed conformation: toroid. Both *b* and *ε*_a_ produce obvious effect on the number of turns of toroid (*N*_t_) and the size of toroid. For the same *b*, the *N*_t_ increases with increase in *ε_a_*, whereas the size of toroid decreases. For the same *ε*_a_, the *N*_t_ decreases with the increase in *b*, whereas the size of toroid increases. It indicates that the attractive monomer–surface interaction plays the role of strengthening the self-attractive interaction of semiflexible polymer.

## Abbreviations

DMC: dynamic Monte Carlo
FENE: finitely extendable nonlinear elastic
MCS: Monte Carlo step
